# Landing on branches in the frog *Trachycephalus resinifictrix* (Anura: Hylidae)

**DOI:** 10.1007/s00359-016-1069-0

**Published:** 2016-01-23

**Authors:** Nienke N. Bijma, Stanislav N. Gorb, Thomas Kleinteich

**Affiliations:** Functional Morphology and Biomechanics, Zoological Institute, Kiel University, Am Botanischen Garten 9, 24118 Kiel, Germany

**Keywords:** Arboreal locomotion, Kinematics, Adhesion, Jumping, Biomechanics

## Abstract

Frogs (Lissamphibia: Anura) are famous for their saltatory or hopping locomotion, which is related to numerous anatomical specialisations that are characteristic for the group. However, while the biomechanics of take-off in frogs have been studied in detail, much less is known on how frogs land after a jump. Besides terrestrial and aquatic species, several lineages of frogs adopted an arboreal lifestyle and especially the biomechanics of landing on challenging, small, and unpredictable substrates, such as leaves or branches, are virtually unknown. Here we studied the landing kinematics of the arboreal frog *Trachycephalus resinifictrix* (Hylidae) on a wooden stick that was used to mimic a small tree branch. We observed two different landing behaviours: (1) landing on the abdomen and (2) attachment with the toes of either the forelimb or the hindlimb. In the latter case, the frogs performed a cartwheel around the stick, while they were only attached by their adhesive toe pads. We estimated the forces that act on the toes during this behaviour to be up to fourteen times the body weight of the animals. This behaviour demonstrates the remarkable adhesive capabilities of the toe pads and the body control of the frogs.

**Electronic supplementary material:**

The online version of this article (doi:10.1007/s00359-016-1069-0) contains supplementary material, which is available to authorized users.

## Introduction

Frogs (Anura) are well known for their saltatory locomotion, which is one of the key characteristics of the group. Anatomical specialisations for jumping have even been identified in the representative of the stem-group anuran *Prosalirus bitis* that dates back to the early Jurassic (Shubin and Jenkins 1995; Jenkins and Shubin 1998). The evolution of frog jumping has received notable attention in the past decades (Gans and Parsons 1966; Emerson 1978; Zug 1985; Kargo et al. 2002; Prikryl et al. 2009; Reilly and Jorgensen 2011; Astley and Roberts 2012; Jorgensen and Reilly 2013; Astley et al. 2013; Astley and Roberts 2014) and provides a popular textbook example in vertebrate comparative biomechanics (Vogel 2003).

Most of these studies, however, focus on the take-off phase and the associated anatomical specialisations of the pelvis and hindlimbs. Much less is known on how frogs actually land after a jump. Generally, there seem to be two mechanisms for landing (Essner et al. 2010): (1) touchdown with the body while the limbs are stretched out and (2) a controlled touchdown with the forelimbs. The latter received particular attention and it has been shown that frogs that land on their forelimbs anticipate the impact and activate their forelimb muscles before touchdown (Gillis et al. 2010, 2014). Nauwelaerts and Aerts (2006) measured the forces during landing and discussed the orientation of the forelimb to dampen the energy of jumps at touchdown. Azizi et al. (2014) further demonstrated that frogs that land on their forelimbs flex their hindlimbs towards the body before landing to align their centre of mass with the orientation of the forelimbs. Limb flexion during the jump in the toad *Bufo marinus* was suggested to be facilitated by an elastic recoil mechanism (Schnyer et al. 2014). In another recent study, the anatomy of the pectoral girdle was described in relation to its three-dimensional movements during landing in a toad (Griep et al. 2013). In all of these studies, landing was observed on a planar surface.

Frogs, however, can be found in numerous terrestrial and aquatic habitats (Duellman and Trueb 1994) and while the more basal frog lineages are mostly living on the ground, several groups independently evolved an arboreal lifestyle (Frost et al. 2006; Wells 2010; Reilly and Jorgensen 2011). Surprisingly, besides a recent study on arboreal frogs walking on thin branches (Herrel et al. 2013), patterns of arboreal locomotion are virtually unknown. Especially the landing behaviour of frogs in an arboreal habitat is expected to be different from that of frogs living on the ground because arboreal species land on narrow and often unpredictable substrates, such as thin branches or leaves. Safe landing seems especially crucial for arboreal frogs, as missing the target can have much more severe consequences than when jumping on the ground. At least, climbing back up to the canopy after a missed jump will be costly for the animals in terms of energy consumption. It seems likely that arboreal frogs show a specialised landing behaviour on narrow substrates that is not exhibited by terrestrial species landing on planar surfaces.

Several lineages of frogs have adhesive toe pads that evolved multiple times within the Anura but show a remarkably high structural similarity among different groups of frogs (Noble and Jaeckle 1928; Emerson and Diehl 1980; Barnes et al. 2013; Drotlef et al. 2015). While these toe pads (or parts of them) can also be present in ground-dwelling species (Noble and Jaeckle 1928; Manzano et al. 2007), they are generally considered to be an adaptation to climbing and consequently climbing is hypothesised to be the main reason why the pads are well developed in arboreal frogs (Barnes 2007). However, besides climbing, these toe pads are likely to play an important role during arboreal landing because they can be used to produce a safe grip and strong damping for these animals at touchdown. The contribution of toe pad adhesion during landing has never been shown before.

**Fig. 1:**
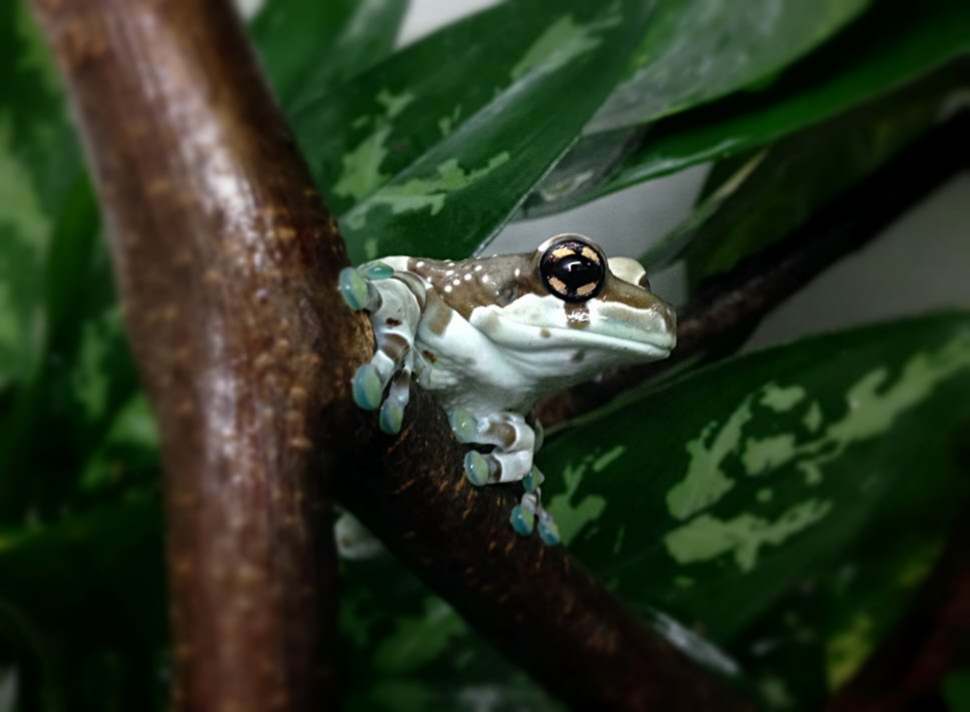
*Trachycephalus resinifictrix* sitting on a *narrow* branch, adhering with its well-developed adhesive toe pads

Here we used high-speed videography and kinematic analysis to observe the landing behaviour of *Trachycephalus resinifictrix* (Anura: Hylidae) on a thin substrate. *T. resinifictrix* is an arboreal species that is native to South America (AmphibiaWeb 2015). These frogs with well-developed toe pads (Fig. 1) originally only occur in primary forests, where they live high up in the canopy (Hödl 1991). The aims of this study were (1) to describe and quantify the movements of *T. resinifictrix* during landing on a narrow stick, (2) to estimate the velocities of the frogs before landing, and (3) to estimate the effectiveness of adhesive pads under the typical behavioural situation and at the natural range of forces that act on the adhesive toe pads during landing in vivo.

## Materials and methods

### Specimens

Four specimens of *Trachycephalus resinifictrix* (Goeldi 1907) were available for this experiment. This species of frog is often kept as a pet and traded under the name Amazon milk frog, but it is also commonly referred to as Mission golden-eyed treefrog (Frank and Ramus 1995; Frost 2015). The specimens were captive bred individuals that we purchased from the local pet trade. The animals ranged from 5.1 to 6.4 cm in snout–vent length (SVL) and weighed between 7 and 19 g. Because the body weight in the animals was highly dependent on food intake and the fullness of the bladder, we weighed the frogs for each experimental trial (Supplementary Table 1). The animals have a bluish coloration with a pattern of beige or brown stripes on their back (Fig. 1), which were used to identify individuals. Two of the animals were identified as mature males with well-developed vocal sacs and nuptial pads. The two other individuals were females, because based on their age they should have reached sexual maturity, but they clearly lacked any male characters. We kept the frogs in a 50 × 50 × 100 cm (width × depth × height) terrarium at a relative humidity of 70–90 %, an ambient temperature of 26–29 °C, and with a 12-h daylight period. The animals were fed twice a week ad libitum with crickets (*Gryllus bimaculatus*).

### Experimental setup

We performed two sets of experiments for which we recorded the movements of the frogs with a high-speed video camera (Photron Fastcam 1024 PCI, Photron Europe Ltd., West Wycombe, Bucks, UK) at 1000 frames per second: (1) free hanging, and (2) landing after a jump. For scaling, we further recorded a ruler that was placed at the same position as the frogs during the experiment with the identical camera settings as during experimental trials. In both experiments, we used a wooden cylindrical stick with a diameter of 1.0 cm as target surface for the frogs. By using a white-light interferometer (New View 6000, Zygo Corporation, Middlefield, CT, USA) we determined the root mean square roughness of this stick to vary between 1.3 and 4.3 µm.

#### Free hanging

We placed the frogs by hand underneath a horizontally oriented wooden stick and allowed them to place one hand on the side face of the stick. We then removed our hands and let the frogs hang free from the stick. Immediately after we removed the hand, the frogs pulled themselves up towards the stick, while we captured the movements of the frog in dorsal view from a perspective perpendicular to the stick.

#### Landing

**Fig. 2:**
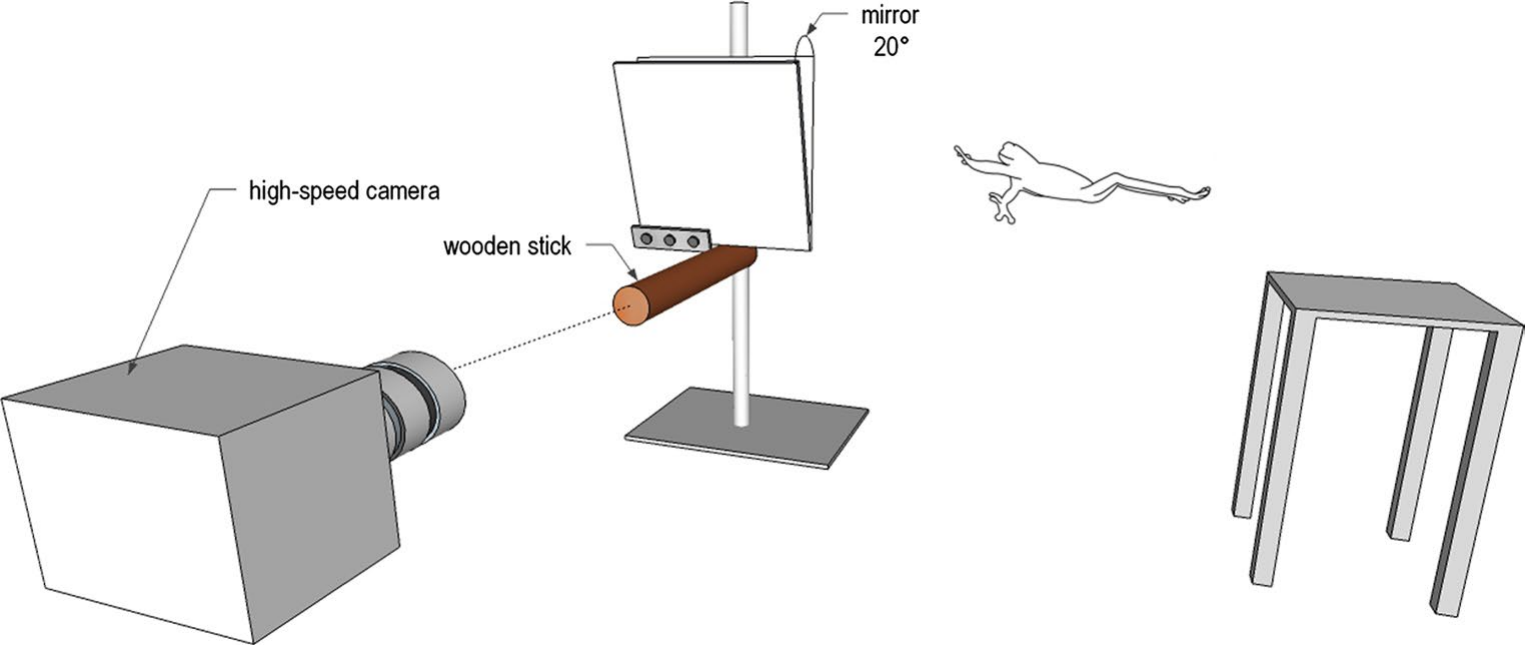
Schematic drawing of the experimental setup for landing trials. A wooden cylindrical stick was placed 25 cm away from a platform that we used to launch the frogs. Platform and target were at a height of 35 cm. A mirror (20 × 20 cm) was mounted with an inclination of 20° to the front to allow for a dorsal view. Using a high-speed video camera, we filmed the frogs while landing on the wooden stick in lateral view

Figure 2 shows the experimental setup used to capture the landing movements of the frogs. We launched the frogs from a Swiss Boy lab jack (Grauer AG, Degersheim, Switzerland), which we adjusted to a height of 35 cm. In a distance of 25 cm, we mounted a wooden stick horizontally in the same height as the lab jack by using a laboratory stand. The wooden stick was facing towards the high-speed video camera with which we recorded the approach by the frogs in lateral view. To identify grip types which the frogs used for landing, we placed a mirror (size: 20 × 20 cm) above the wooden stick at an angle of 20°. To make the frogs jump, we placed them by hand on the lab jack and gently tapped their hind legs. For each individual, we recorded ten experimental trials.

### Data analysis

**Fig. 3:**
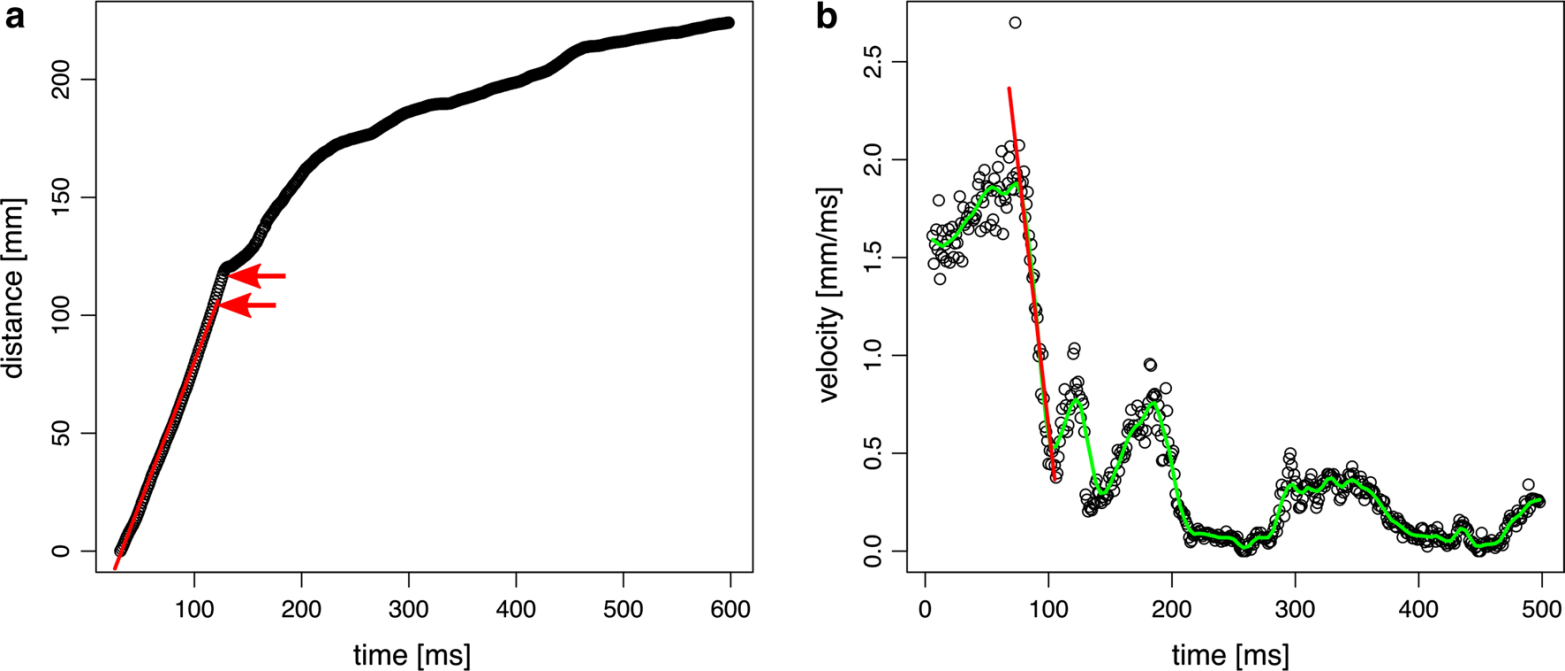
Regression analysis to calculate mean velocities during the approach (**a**) and negative accelerations after contact (**b**). The *red lines* show the regression function; the *green line* in **b** shows a locally weighted polynomial regression of the raw data (lowess function in R stats package), which is used for visual inspection of the graph only; calculations are based on the raw data. In the example shown here (frog ID2, trial 1), the frog leaped over the target and reached backwards with its forelimb to make contact with the stick. **a** The velocity of the frog is calculated based on the flight path (distance over time) until the first contact with the stick is reached (*lower arrow*). The frog maintains most of its forward speed until the arm is completely stretched (distance between both *arrows*) and then starts to decelerate. **b** The deceleration during landing is calculated from the moment of the first attachment with the stick, until the frog gets redirected on a* circular path*. After slowing down to its minimum velocity, the frog accelerates and decelerates in an oscillating movement

To analyse the high-speed video recordings, we used the landmark tracking plug-in MTrackJ (Meijering et al. 2012) for the image analysis software Image J 1.48 (available at http://www.imagej.nih.gov/ij/). For each experiment, we adjusted the pixel to cm ratio based on the video recordings of the ruler. Each landing video sequence was then analysed frame by frame, which at a recording frequency of 1000 frames per second resulted in a temporal resolution of 1 ms. For the experimental trials in which the frogs were free hanging at the stick, we tracked the position of the toe pads attached to the stick, to identify sliding movements. However, it turned out that in all experimental trials, the toe pads remained stationary once they are attached and sliding was never observed. For landing trials we traced the position of the rostral tip of the nasal capsule and the position of the toes in lateral view. MTrackJ outputs the *x*- and *y*-coordinates of each track of landmarks, as well as the travelled distances, and velocities over time. The velocity data in MTrackJ corresponds to the travelled distance per frame, i.e. per ms. For further analysis, we imported these data into the statistical computation software R 3.1.1 (available at http://www.r-project.org). For each trial we calculated a linear regression of distance over time for times ranging from the onset of the recorded videos until the frogs came in contact with the target. The slope of this regression is a measure of the average velocity of the frogs during the approach, which is different from the frame-by-frame velocities that MTrackJ calculated (Fig. 3a; for regression statistics see Supplementary Table 1). Further, for trials in which the frogs were attached to the wooden stick with their forelimbs, we further calculated a linear regression of changes in velocity over time for the deceleration phase, i.e. after the frogs made contact with the stick but before oscillation movements occurred (Fig. 3b; Supplementary Table 2). The slope of this regression is a measure for the deceleration the frogs experienced, which multiplied by the mass of the animals gives an estimation of the forces acting on the toe pads during this phase of landing.

## Results

### Free hanging

To estimate the performance of the toe pads in *T. resinifictrix*, we first tested whether the frogs were able to support their body weight if hanging on horizontally oriented cylindrically shaped branches using their individual toes. We found that all specimens tested (*N* = 4) were able to carry their own body weight on only two digits on either forelimb, if the contact was made by digits three and four. In one case, the frog used only one toe pad (digit three) for free hanging. Once attached to the stick, the toes were not moving or slipping down. However, usually the frogs started to pull themselves up or brought the second forelimb into contact immediately after the onset of the experiment (Supplementary Video 1). Thus, the time of free hanging was limited and ranged from 51 to 277 ms (*N* = 7; average free hanging time: 141 ± 90 ms). In one case, the frog hung for approximately 5 s, which exceeded the time we were able to record with our experimental setup (1.54 s) and thus this measurement had to be excluded from the calculation of the average free hanging time.

### Landing

To study the kinematics of landing on a thin substrate, we had *T. resinifictrix* jump onto a horizontally oriented wooden stick with 1 cm diameter and filmed a total of 40 approaches in lateral view with a high-speed camera at 1000 frames per second. We never observed frogs missing the wooden stick in our experiment nor did we see trials where the animals failed to arrest the jumps. Kinematic analysis of the video sequences revealed that the frogs approached the stick with an average velocity of 1.34 ± 0.19 m/s (*N* = 40; Supplementary Table 1). We found that the frog ID1 was significantly slower during the approach than the animal ID2 but we did not observe further statistically significant differences between the animals (one-way ANOVA in combination with Tukey’s honest significant difference test; *F* = 3.38,* df*1 = 3,* df*2 = 36, level of significance *p* < 0.05). While the frogs are in the air, their limbs are widespread to increase the reach between their arms and legs, respectively, and also to stabilise their flight.

**Fig. 4:**
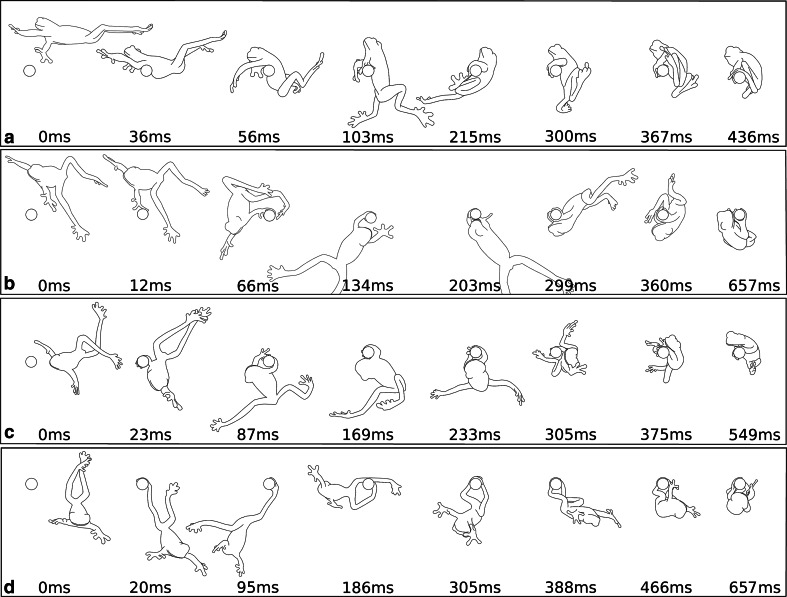
Representative trajectories for the two different landing strategies on narrow substrates. Jumps are from *right* to *left*. Landing on the abdomen (**a**) and landing by using their adhesive toe pads on their limbs (**b**–**d**). The frogs can use their forelimbs (**b**, **c**) or hindlimbs (**d**) for landing. If the frogs land by using their limbs, they differ by a variation of the position of the body relative to the target: **b** leaping over the target, reaching towards the stick backwards. **c**, **d** Jumping too short and reaching the target by moving the limb forwards

After the aerial phase, we observed two different landing strategies (Fig. 4): (1) landing on the abdomen (i.e. a belly-flop) (*N* = 20; Fig. 4a; Supplementary Video 2), or (2) landing by using the adhesive toe pads on the limbs (*N* = 20; Fig. 2b–d). Either the toe pads of the forelimb (*N* = 16; Fig. 4b, c; Supplementary Video 3) or the hindlimb (*N* = 4; Fig. 4d; Supplementary Video 4) were used for attachment to the stick. Further, we found variation in the position of the frogs relative to the target during landing with the limbs. The frogs either leaped over the target and reached backwards with the trailing forelimb (*N* = 8; Fig. 4b; Supplementary Video 3) or descended before they reached the target in which case they reached forwards with the leading forelimb (*N* = 8; Fig. 4c; Supplementary Video 5) or hindlimb (*N* = 4; Fig. 4d; Supplementary Video 4).

If the frogs used the limbs for attachment, they always performed a yaw movement before touchdown to orient their limbs towards the target; in some cases this behaviour was also observed during abdominal landing and helped the frogs to orient themselves parallel to the target. Alternatively, if landing on the abdomen, the frogs did also touchdown in perpendicular orientation to the target in which case the body folded around the stick, which decelerated the frog immediately (Fig. 4a). After impact, the frogs immediately grabbed the stick with their adhesive toe pads to hold on to the target.

In experimental trials in which the frogs touched the target stick with one of their limbs to initiate landing, the toe pads in contact did immediately stick to the target without sliding along the substrate. During these trials, the frogs first proceeded on their parabolic jump trajectory after one limb was in contact with the target until this limb was fully stretched. Then, due to the fixation of the animal at the toe pads in contact, the frogs were redirected on a circular path and performed a cartwheel movement (Fig. 4b–d; Supplementary Videos 3–5). In some cases, this cartwheel allowed the frogs to land on top of the stick; in most cases, however, the frogs started to oscillate like a pendulum mounted to the stick. During this oscillating motion, the frogs pulled themselves up towards the stick. Generally after one or two swings, the frogs brought a second limb into contact with the target to manoeuvre themselves on top of the stick.

We identified eight different grip types on the forelimb, which the frogs used to cling themselves to the target while landing (Table 1). In total, we observed 27 landings in which the forelimbs were used for deceleration, either by direct contact or after the frogs touched down on the abdomen; in nine cases, the frogs were stopped by their abdomen without notable attachment of the forelimbs. We found that generally all digits on the forelimb can be used for attachment. However, from the 27 landings on which the forelimb was involved (including cases where the abdomen touches down first), in 13, i.e. almost 50 %, of the observed trials only digits D3 and/or D4 were used (Table 1). In cases in which the hindlimb first held onto the target, we found two different grip types involving digit D5 (*N* = 1) or digits two, three, four, and five simultaneously (*N* = 3) (Table 1).

**Table 1:** Grip types observed during landings of T. resinifictrix on a wooden stick

Landings for which toes were used (*N* = 31)	Frequency of grip type (D = digit; *D5 only at hindlimb)									
	D2	D4	D12	D23	D24	D34	D123	D234	D5*	D2345*
Abdomen (*N* = 11)		5 (45 %)		2 (18 %)		2 (18 %)	2 (18 %)			
Forelimb										
Reaching forward (*N* = 8)		1 (12.5 %)	1 (12.5 %)	2 (25 %)	1 (12.5 %)		1 (12.5 %)	2 (25 %)		
Reaching backward (*N* = 8)	2 (25 %)	1 (12.5 %)		1 (12.5 %)		4 (50%)				
Hindlimb										
Reaching forward (*N* = 4)									1 (25 %)	3 (75 %)

To estimate the forces that act on the toes of the animals when the frogs are only attached by one of their fore- or hindlimbs during landing, we calculated the deceleration of the frogs by linear regression of velocity over time. In eight cases the statistical support for the linear regression was poor (*p* > 0.05; Supplementary Table 2) and these were excluded from the analysis. In the remainder of the trials, deceleration ranged from 6.1 m/s^2^ (specimen ID2 leaping over the target and reaching backwards with digit D4 of the forelimb) to 141.6 m/s^2^ (specimen ID3 reaching forward with the forelimb and making contact with digit D4) and was on average 47.3 ± 42.4 m/s^2^ (*N* = 12, Table 2). This corresponds to up to 14.4 times the gravitational acceleration (calculated from 141.6/9.81 = 14.4). Calculation of deceleration values times the mass of the frogs gives an estimate of the forces that act on the toe pads during landing. We found that toe pads withstand forces between 0.11 and 1.27 N (on average 0.55 ± 0.40 N, *N* = 12, Table 2), which corresponds to 62 % and up to 1443 % of the body weight of the animals (Table 2).

**Table 2:** Calculated negative accelerations and forces experienced by *T. resinifictrix* during attachment with the forelimb

Specimen ID (trial #)	Mass of specimen (g)	Acceleration (m/s^2^)	Acceleration per gravity acceleration	Force (*N*)	Force (% body weight)
2 (1)	17	−19.31	−2.0	−0.328	196.7
2 (2)	17	−13.72	−1.4	−0.233	139.7
2 (4)	17	−77.00	−7.8	−1.309	784.9
2 (5)	17	−20.55	−2.1	−0.349	209.3
2 (10)	18	−6.10	−0.6	−0.110	62.3
3 (5)	9	−141.56	−14.4	−1.274	1443.0
3 (6)	7	−109.64	−11.2	−0.767	1116.9
4 (1)	13.5	−52.02	−5.3	−0.702	530.1
4 (2)	11	−28.35	−2.9	−0.312	289.1
4 (3)	11	−52.57	−5.4	−0.578	535.6
4 (5)	14	−33.08	−3.4	−0.463	337.1
4 (7)	14	−13.38	−1.4	−0.187	136.2

## Discussion

For the arboreal frog *T. resinifictrix,* safe landing is essential since they are living in heights of more than 10 m (Hödl 1991; Honigs et al. 2014). A failure in landing could be fatal or at least a loss of height would increase the stresses on the locomotor apparatus for landing (Günther et al. 1991). Further, it would be energetically very costly for these animals to climb back. The target (i.e. a branch or a leaf) itself might change its position while the frog is in the air (e.g. due to wind) or at impact of the frog, which makes it challenging for the animals to estimate their jumping trajectories a priori. We found that *T. resinifictrix* overcomes these challenges by showing a high plasticity on the choice of a landing strategy. Strikingly, in cases where the limbs are used, the frogs performed a partial cartwheel around the landing stick, which demonstrates the excellent adhesiveness of their toe pads and the body control of these animals. To our knowledge, a similar behaviour has never been documented in frogs before.

It has been previously shown that the landing strategies of frogs on plain surfaces can differ significantly between species. This is in contrast to the take-off phase that appears to be largely conserved across anurans (Emerson and De Jongh 1980; Zug 1985; Essner et al. 2010). More basal frogs, like *Ascaphus montanus*, tend to land on their abdomen, although depending on the angle of attack during landing the fore or hind-limbs can touch the surface first (Essner et al. 2010). Toads (Bufonidae) and so-called true frogs (Ranidae) in contrast, land balanced and stable with their forelimbs first (Nauwelaerts and Aerts 2006; Griep et al. 2013; Azizi et al. 2014; Gillis et al. 2014). Here, when landing on a stick, we observed both landing mechanisms (on the abdomen and landing with the forelimbs) in *T. resinifictrix*. In contrast to toads, where the limbs are moved close to the body before impact to ensure stable landing (Azizi et al. 2014), *T. resinifictrix* always stretches its limbs out during the jump. This is similar to the behaviour of *A. montanus*, which during landing on the ground results in a belly flop (Essner et al. 2010). However, in the arboreal scenario, a flat body posture with stretched out limbs will stabilise the flight and also increases the chances for the frog to make contact with a target.

Both strategies, landing on the abdomen versus attaching with the toe pads on the forelimbs or hindlimbs, have advantages and disadvantages for the frogs. By landing on the abdomen, the exact estimation of the target position in space seems less critical as the chances of missing the target are minimised. Further, overshooting the target is less likely, as the abdomen of the frog will immediately stop the flight, while if only the toes are in contact with the target, the frogs proceed on their trajectory. However, during abdominal landing, the abdomen has to dissipate all of the frog’s kinetic energy, which could potentially cause harm to visceral organs. If the first contact is established with the adhesive toe pads, however, kinetic energy will be dissipated during the cartwheel movement. This method demands for strong adhesion at the toe pads and for a placement of the toes on the target.

Here we demonstrated that even a single toe pad is sticky enough to hold the body weight of the frogs on the curved substrate. It was previously shown that shearing, respectively frictional forces (i.e. forces acting parallel to the contact surface), are important to secure the attachment of tree frog toe pads (Barnes et al. 2006, 2008; Endlein et al. 2013). Even in the case of landings, where the forces acting on the toe pads are beyond the body weight of the animals, we never observed sliding movements of the toe pads after the limbs were in contact with the surface. Thus, in vivo friction between one or two toe pads and the target surface is suitable to hold the entire animal during deceleration. The forces during deceleration, which we estimated herein (up to fourteen times the body weight of the frog), are notably higher than the shearing forces previously reported for tree frog toe pads. These forces were reported to be in the range of the body weight of the frogs in animals with a comparable size to *T. resinifictrix* (Barnes et al. 2006). One part of this variation might be explained by differences in the target surface. In previous experiments, the frogs were placed on a smooth Perspex thermoplastic sheet (Barnes et al. 2006) that probably has a lower frictional coefficient in contact with the frogs’ toe pads than the rough wood surface. Further, Hanna and Barnes (1991) reported that measured frictional forces on toe pads increased if the tilting platform that was used for force measurements was moved rapidly. So after the contact between the toe pads and the target is established, the viscoelasticity of the toe pads will allow for stronger frictional forces at high velocities. However, during landing, viscoelasticity of the toe pads might actually cause resistance during contact formation. In any case, the contribution of the pad viscoelasticity to the highly dynamic processes of jump start and landing should be considered in future experimental studies.

**Fig. 5:**
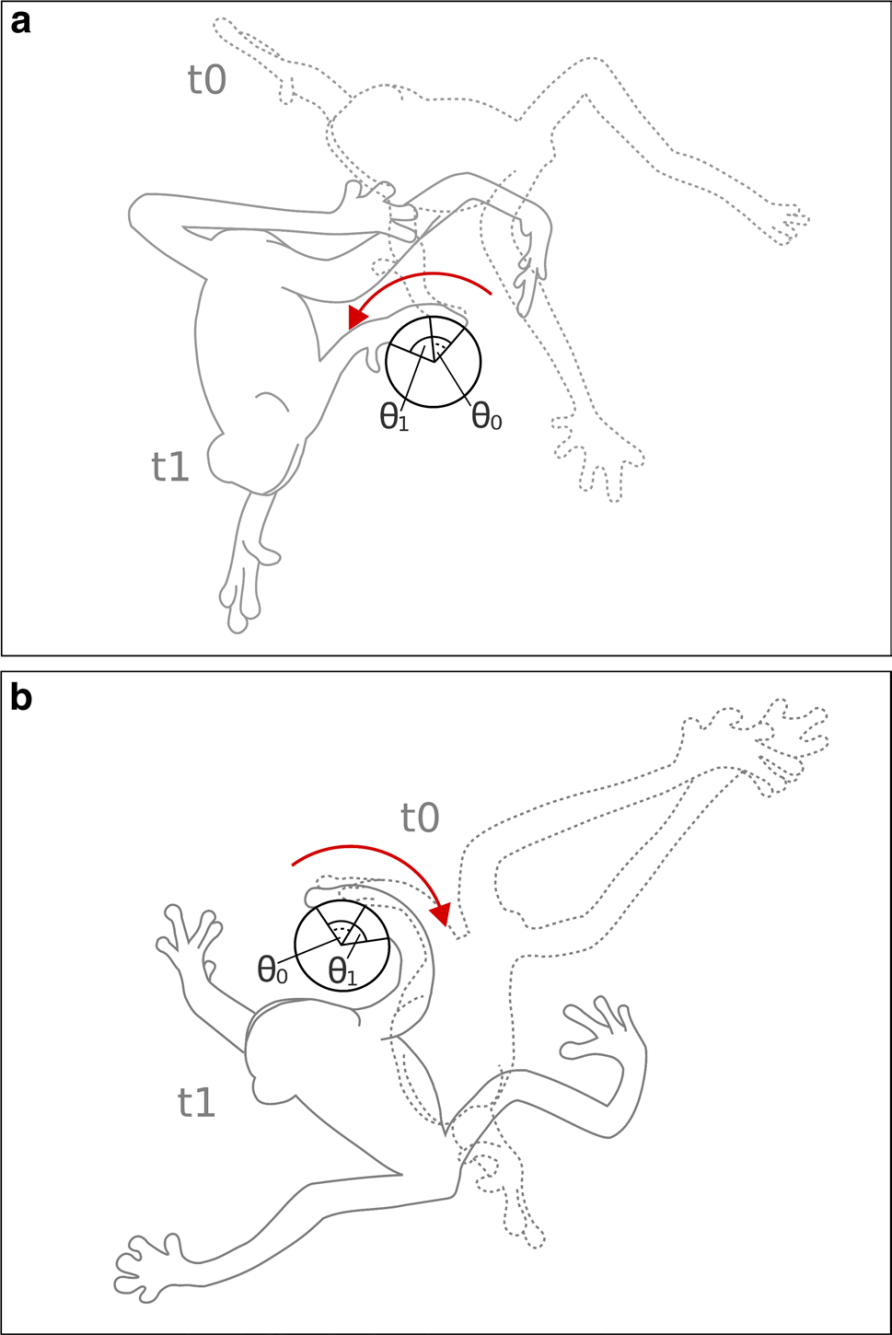
Scheme demonstrating the increase of friction during landing by wrapping fingers around the target stick. The *dashed outline* of a frog (*t*_0_) shows the early stage of contact during landing. The contact angle *θ*_0_ between stick and frog increases (*θ*_1_) as the frog continues its trajectory (*t*_0_). The *red arrow* represents the direction of this enlargement

Besides differences in frictional coefficients due to the difference of experimental setups and the dynamics of the movement in the previous publications and the present study, the frogs in our experiment seemed to further maximise frictional forces by placing the attached toes on the face of the target that is opposite to their body and wrapping their digits around the stick (Figs. 4, 5; Supplementary Videos 3 and 5). The Capstan Equation demonstrates the relationship between friction in the contact area and the hold force:

1 $$F_{1} = \, F_{0} * \, e^{\mu \theta }$$

with *F*_1_ as the loading force (i.e. the body weight of the frog), *F*_0_ as the holding force (i.e. the force acting on the toe), *µ* as the frictional coefficient between two contacting solids, and *θ* as the angle that is swept by the holding limb. Thus, wrapping the limb around the stick (i.e. enlarging the angle *θ*) has a similar effect as an increase in the frictional coefficient and allows for higher loading forces with the same amount of holding force (Jung et al. 2008). In *T. resinifictrix*, after the toes are in contact with the target, the frogs still continue to move on the jumping trajectory before deceleration. During this phase, however, the limb in contact already wraps around the stick, which increases the effective frictional forces (Fig. 5). Based on the high-speed videos we recorded herein, angle *θ* appears to be in the range of 180°, which corresponds to *π* if measured in radians. The effective frictional coefficient between the limbs in contact and the target stick will thus be increased more than three times due to wrapping of the fingers around the stick. We also observed a similar behaviour during the free hanging experiment described herein. There, the frogs also increased the wrapping angle by adapting the finger to the shape of the stick.

We further found that the frogs perform a yaw movement of their bodies to the left or the right during the approach before contact with the target. This yaw is always observed in cases where the frogs touch down with the limbs first and only occasionally during abdominal landing. Because we only observed landings from a lateral view, we were not able to follow the three-dimensional trajectory of the frogs after take-off and during the approach. Thus, the extent and control of this yaw remain cryptic. Besides an active control of the trajectory during the flight phase, this yaw might be caused by an asymmetrical jump, which was previously observed in primates (Jouffroy and Gasc 1974; Günther et al. 1991). In either way, this yaw movement seems to play an important role in the choice of the preferred landing strategy. For landing with the limbs and the cartwheel movement thereafter, it might be beneficial if the body is oriented parallel to the target stick before impact to improve the grip with the toe pads. In contrast, for abdominal landing it can be advantageous to touch down with the entire width of the abdomen.

We noticed individual differences between the frogs and some of these might be related to the body weight of the animals. The two larger specimens (ID1 and ID2) more often performed abdominal landings compared to the smaller animals (Supplementary Table 1). Ontogenetic and scaling effects on the choice of landing strategies in frogs were not in focus here and will require further research. Further, other than scaling, experience, training level, and individual preference might all influence the choice of the landing method.

Besides stretching the limbs out, *T. resinifictrix* further increased the chances for a successful contact by exhibiting a diversity of grip types that can be applied. In our experiment, we observed eight different combinations of digits that were brought into contact with the target. For comparison, in a previous study on arboreal locomotion in frogs, Herrel et al. (2013) described only three main grip types for walking on thin branches. During landing after a jump, a precise placement of the toes is expected to be more challenging than during walking. Thus, *T. resinifictrix* seems to use any digit that is in a favourable position for contact, with no clear preference. However, similar to the observation made by Herrel et al. (2013), digit D1 is rarely used. Further, Herrel et al. (2013) described that digits D3 and D4 are usually used by frogs walking on horizontally oriented substrates, which might be related to our results herein where we found that grip types involving these two digits are used most frequently for landing on a horizontal target.

In summary, we showed that *T. resinifictrix* employs two different strategies for successful landings on challenging surfaces. The choice between these two landing strategies appears to happen during the aerial phase and is influenced by the body position relative to the target. If and how this position is actively adjusted remains to be resolved in future research projects. At contact, the toe pads of the frogs ensure an instant and strong contact to secure the frogs at the target surface. The adhesion of the toe pads is strong enough to allow the frogs to cling themselves to the target with only a few toes attached, which results in the spectacular movements we reported herein.

## Electronic supplementary material

Below is the link to the electronic supplementary material.

Supplementary Video 1: Exemplary trial of a free hanging experiment (MOV 5899 kb)

Supplementary Video 2: Exemplary trial of a landing experiment at which the frog lands on the abdomen (MOV 7496 kb)

Supplementary Video 3: Exemplary trial of a landing experiment at which the frog leaps over the target and reaches backward (MOV 10366 kb)

Supplementary Video 4: Exemplary trial of a landing experiment at which the frog attaches a hindlimb first (MOV 12473 kb)

Supplementary Video 5: Exemplary trial of a landing experiment at which the frog descends before the target and reaches forward (MOV 8694 kb)

Supplementary Table 1: Raw data for observed landing movements and calculation of velocities during the approach. Supplementary Table 2: Results of regression analysis to calculate negative accelerations after the toe pads attached to the target (PDF 145 kb)
