# Supplementary material for: Landing on branches in the frog Trachycephalus resinifictrix (Anura: Hylidae)
Source: J Comp Physiol A Neuroethol Sens Neural Behav Physiol. 2016 Jan 23;202:267–76. doi: 10.1007/s00359-016-1069-0 (PMC4819504; doi:10.1007/s00359-016-1069-0)
Supplement: Supplementary file 6 — Supplementary Table 1: Raw data for observed landing movements and calculation of velocities during the approach. Supplementary Table 2: Results of regression analysis to calculate negative accelerations after the toe pads attached to the target (PDF 145 kb) [file 359_2016_1069_MOESM6_ESM.pdf]

Supplementary Information

Landing on branches in the frog *Trachycephalus resinifictrix* (Anura: Hylidae)

Nienke N. Bijma, Stanislav N. Gorb, and Thomas Kleinteich

Kiel University, Functional Morphology and Biomechanics, Am Botanischen Garten 1-9, 24118 Kiel, Germany

Supplementary Table 1: Raw data for observed landing movements and calculation of velocities during the approach

Specimen ID	Trial #	Mass [g]	Landing behaviour	Grip-type	Linear regression of travelled distance over time for approach, *** shows high statistical significance ($p < 0.001$)						
					Velocity [m/s]	Std error [m/s]	T value	R^2	F value	DF1	DF2
1	1	17	Abdomen	-	**1.33**	0.01	245.00***	0.999	60040***	1	73
1	2	17	Abdomen	D4 left	**1.21**	0.01	187.47***	0.997	35140***	1	89
1	3	19	Abdomen	-	**1.16**	0.01	186.94***	0.998	34950***	1	67
1	4	19	Forelimb - backwards	D34 left	**1.41**	0.00	342.10***	>0.999	117000***	1	50
1	5	19	Abdomen	D4 right	**1.31**	0.00	516.80***	>0.999	267100***	1	59
1	6	18	Abdomen	-	**1.16**	0.01	234.95***	0.999	55200***	1	63
1	7	19	Abdomen	D34 right	**1.24**	0.00	449.71***	>0.999	202200***	1	65
1	8	19	Forelimb - backwards	D34 right	**1.10**	0.00	291.74***	0.999	85110***	1	75
1	9	19	Abdomen	D23 right	**1.08**	0.01	181.90***	0.999	33100***	1	46
1	10	19	Abdomen	-	**1.43**	0.01	176.80***	0.998	31250***	1	62
2	1	17	Forelimb - backwards	D2 left	**1.50**	0.01	237.80***	0.999	56570***	1	66
2	2	17	Forelimb - backwards	D34 left	**1.41**	0.00	378.90***	>0.999	143600***	1	48
2	3	17	Abdomen	D4 left	**2.03**	0.01	286.00***	0.999	81800***	1	49
2	4	17	Forelimb - backwards	D2 left	**1.36**	0.01	233.73***	0.999	54630***	1	52
2	5	17	Forelimb - backwards	D23 left	**1.28**	0.00	384.50***	>0.999	147900***	1	56
2	6	16	Abdomen	D34 right	**1.47**	0.01	171.09***	0.999	29270***	1	41
2	7	16	Abdomen	D123 right	**1.33**	0.01	151.91***	0.998	23080***	1	47
2	8	17	Abdomen	D4 left D123 right	**1.43**	0.00	332.20***	>0.999	110400***	1	32
2	9	16	Abdomen	-	**1.29**	0.00	636.50***	>0,999	405200***	1	76
2	10	18	Forelimb - backwards	D4	**1.65**	0.01	324.60***	>0.999	105300***	1	28
3	1	7	Abdomen	-	**1.55**	0.01	143.24***	0.998	20520***	1	49
3	2	7	Hindlimb	D5 foot right	**1.43**	0.01	158.60***	0.997	25160***	1	64
3	3	7	Abdomen	-	**1.13**	0.01	143.60***	0.997	20620***	1	64
3	4	9	Forelimb - forward	D123 right	**1.59**	0.02	107.94***	0.996	11650***	1	50
3	5	9	Forelimb - forward	D4 right	**1.69**	0.02	88.84***	0.992	7893***	1	60
3	6	7	Forelimb - forward	D23right; D123 left	**1.45**	0.01	102.71***	0.995	10550***	1	55
3	7	7	Hindlimb	D2345 foot right	**1.44**	0.01	141.10***	0.998	19910***	1	46
3	8	10	Forelimb - forward	D234 left	**1.32**	0.01	124.06***	0.996	15390***	1	56
3	9	12	Hindlimb	D2345 foot right	**1.15**	0.01	100.96***	0.992	10190***	1	81
3	10	12	Abdomen	-	**1.42**	0.01	298.50***	>0.999	89130***	1	46
4	1	13.5	Forelimb - forward	D23 right	**1.27**	0.00	305.60***	0.999	93390***	1	81
4	2	11	Hindlimb	D2345 foot right	**1.47**	0.01	125.48***	0.995	15750***	1	81
4	3	11	Forelimb - forward	D12 right	**1.60**	0.00	486.00***	>0.999	236200***	1	75
4	4	15	Abdomen	-	**1.48**	0.01	219.30***	0.999	48100***	1	38
4	5	14	Forelimb - forward	D24 right	**1.44**	0.01	272.36***	0.999	74180***	1	53
4	6	14	Abdomen	D23 right	**1.37**	0.01	215.19***	0.998	46310***	1	80
4	7	14	Forelimb - backwards	D34 left	**1.21**	0.00	399.50***	>0.999	159600***	1	74
4	8	14	Abdomen	D4 right	**1.26**	0.01	137.72***	0.996	18970***	1	74
4	9	14	Forelimb - forward	D234 right	**1.70**	0.01	244.70***	0.999	59890***	1	61
4	10	14	Abdomen	-	**1.44**	0.01	282.39***	0.999	79740***	1	66

Supplementary Table 2: Results of regression analysis to calculate negative accelerations after the toe pads attached to the target.

Specimen ID	Trial #	Linear regression of velocity over time after contact; asterisks indicate statistical significance; *** $p < 0.001$, ** $p < 0.01$, * $p < 0.5$							
		Acceleration [m/s^2]	Acceleration [G]	Std error [m/s^2]	T value	R^2	F value	DF1	DF2
1	4	-3.17	-0.32	0.01	-0.25	-0.058	0.06	1	16
1	8	-23.63	-2.41	0.02	-1.25	0.102	1.57	1	4
2	1	-19.31	-1.97	0.00	-10.72***	0.695	115.00***	1	49
2	2	-13.72	-1.40	0.00	-9.66***	0.667	93.24***	1	45
2	4	-77.00	-7.85	0.02	-3.87**	0.635	14.94**	1	7
2	5	-20.55	-2.10	0.01	-4.21***	0.41	17.70***	1	23
2	10	-6.10	-0.62	0.00	-3.43**	0.246	11.78**	1	32
3	2	-7.08	-0.72	0.01	-0.8	-0.034	0.64	1	10
3	4	-13.93	-1.42	0.03	-0.56	-0.108	0.32	1	6
3	5	-141.56	-14.43	0.01	-11.20***	0.919	125.50***	1	10
3	6	-109.64	-11.18	0.02	-4.77***	0.665	22.79***	1	10
3	7	-135.90	-13.85	0.03	-4.06	0.838	16.46	1	2
3	8	-94.63	-9.65	0.06	-1.68	0.268	2.84	1	4
3	9	-3.85	-0.39	0.01	-0.4	-0.118	0.16	1	7
4	1	-52.02	-5.30	0.01	-4.17**	0.598	17.38**	1	10
4	2	-28.35	-2.89	0.00	-23.91***	0.899	571.90***	1	63
4	3	-52.57	-5.36	0.01	-4.95**	0.746	24.50**	1	7
4	5	-33.08	-3.37	0.01	-7.33***	0.687	53.66***	1	23
4	7	-13.38	-1.36	0.00	-5.48***	0.42	30.01***	1	39
4	9	-19.26	-1.96	0.01	-1.45	0.074	2.11	1	13

Supplementary Video 1: Exemplary trial of a free hanging experiment.

Supplementary Video 2: Exemplary trial of a landing experiment at which the frog lands on the abdomen.

Supplementary Video 3: Exemplary trial of a landing experiment at which the frog leaps over the target and reaches backward.

Supplementary Video 4: Exemplary trial of a landing experiment at which the frog attaches a hindlimb first.

Supplementary Video 5: Exemplary trial of a landing experiment at which the frog descends before the target and reaches forward.
